# 

**Published:** 1873-11

**Authors:** 


Vol. III. NOVEMBER, 1873. No. 11.

THE

### SOUTHERN MEDICAL RECORD

A MONTHLY JOURNAL OF

### PRACTICAL MEDICINE.

EDITED BY

T. S. POWELL, M. D.,

AND

W. T. GOLDSMITH, M. D.

*"QUICQUID PRÆCIPIES ESTO BREVIS."*

Atlanta, Georgia.

ECONOMICAL BOOK & JOB PRINTING HOUSE.

V. P. SISSON & CO., PROPRIETORS.

*1873.*

TERMS: $2.50 PER ANNUM, IN ADVANCE. SINGLE NUMBER, 25 CENTS.

